# Complete Recovery of Iatrogenic Radial Nerve Palsy in a Child: A Case Report

**DOI:** 10.7759/cureus.108520

**Published:** 2026-05-08

**Authors:** Sushil Mankar, Rahul H Sakhare, Vijay D Surve, Pallav P Agrawal, Darshan Sharma

**Affiliations:** 1 Orthopaedics and Traumatology, N. K. P. Salve Institute of Medical Sciences and Research Centre and Lata Mangeshkar Hospital, Nagpur, IND; 2 Orthopaedics, N. K. P. Salve Institute of Medical Sciences and Research Centre and Lata Mangeshkar Hospital, Nagpur, IND

**Keywords:** closed reduction internal fixation, iatrogenic radial nerve injury, k-wire, supracondylar humeral fracture, wrist drop

## Abstract

Supracondylar humerus fractures are among the most common paediatric injuries. Although neurologic complications are often seen in these fractures, iatrogenic nerve injuries following closed reduction and percutaneous pinning remain relatively uncommon. Radial nerve palsy is particularly rare in this context, and most cases represent neuropraxic injuries that recover spontaneously. This report describes a rare case of iatrogenic high radial nerve palsy following closed reduction and K-wire fixation of a comminuted supracondylar humerus fracture in a child, with complete functional recovery under conservative management. An eight-year-old boy sustained a comminuted supracondylar humerus fracture after a fall on an outstretched hand. Preoperative neurovascular examination was normal. The patient underwent closed reduction and crossed K-wire fixation, requiring multiple reduction attempts due to fracture comminution. Postoperatively, he developed wrist and finger drop, loss of thumb abduction, and dorsal hand hypoesthesia, consistent with high radial nerve palsy, despite intact distal perfusion and normal postoperative radiographs. Conservative management with close follow-up was initiated. Early neurological improvement was observed at four to six weeks, with radiographic fracture union and K-wire removal at six weeks. By eight weeks, the patient demonstrated complete recovery of wrist extension, thumb abduction, and full elbow range of motion. Radial nerve injury in supracondylar fractures is typically due to traction or entrapment by fracture fragments. Iatrogenic injuries most commonly occur with repeated manipulations during difficult reductions or pin placement. Most such injuries are neuropraxias and recover without surgical intervention. Current evidence supports conservative management unless neurological improvement is absent. To conclude, this case highlights that iatrogenic radial nerve palsy following closed reduction and K-wire fixation, although rare, is usually transient. Thorough clinical assessment and close follow-up are essential. Conservative management can result in complete neurological recovery, and early surgical exploration is generally unnecessary unless deficits persist beyond the expected recovery timeframe.

## Introduction

Supracondylar humerus fractures are among the most common paediatric upper limb injuries, accounting for approximately 15% of all paediatric fractures, with the majority occurring in the first decade of life [1].

Iatrogenic nerve injury following closed reduction and percutaneous pinning - the standard surgical treatment for displaced fractures - represents a distinct and less frequently discussed subset of these complications. The radial nerve, and specifically its deep branch, is considered particularly vulnerable during lateral pin placement; however, high radial nerve palsy following Closed reduction internal fixation remains exceptionally rare in the published literature, with only isolated case reports describing this presentation [2]. Neurovascular complications are well-recognised, affecting approximately 10-15% of cases, with the anterior interosseous nerve and brachial artery most frequently implicated [3]. Compartment syndrome, while serious, occurs in only 2-3% of cases [4].

Surgeons may not anticipate high radial nerve palsy as a postoperative complication, potentially delaying recognition and management. We present the case of an eight-year-old male who developed high radial nerve palsy following CRPP of a comminuted supracondylar humerus fracture, with the aim of raising clinical awareness and contributing to the limited evidence base surrounding this uncommon iatrogenic injury.

## Case presentation

**Figure 1:**
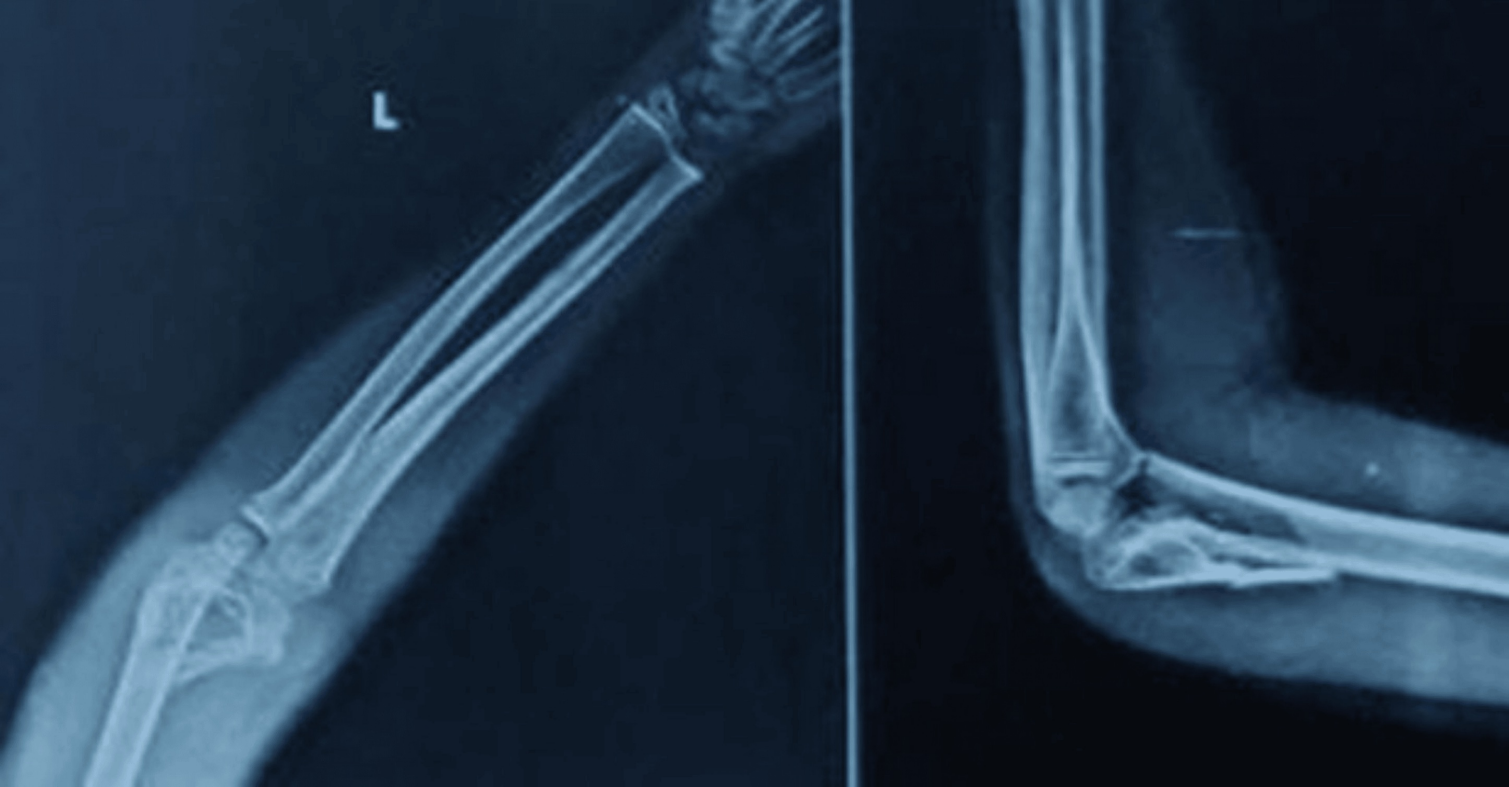
Radiograph of the left elbow. Anteroposterior and mediolateral views, suggestive of a comminuted displaced supracondylar humerus fracture.

An eight-year-old boy was brought by his parents to our emergency department with complaints of pain and deformity of the left elbow for eight hours. The patient had a fall on an outstretched hand while playing. On examination, there was pain and swelling in the right elbow, and the elbow movements were restricted due to pain. There were no open wounds, and the neurological examination was normal, and the distal vascularity of the upper limb was intact with good hand perfusion. Radiographs of the right elbow in anteroposterior and mediolateral views showed a comminuted supracondylar humerus fracture (Figure 1).

**Figure 2:**
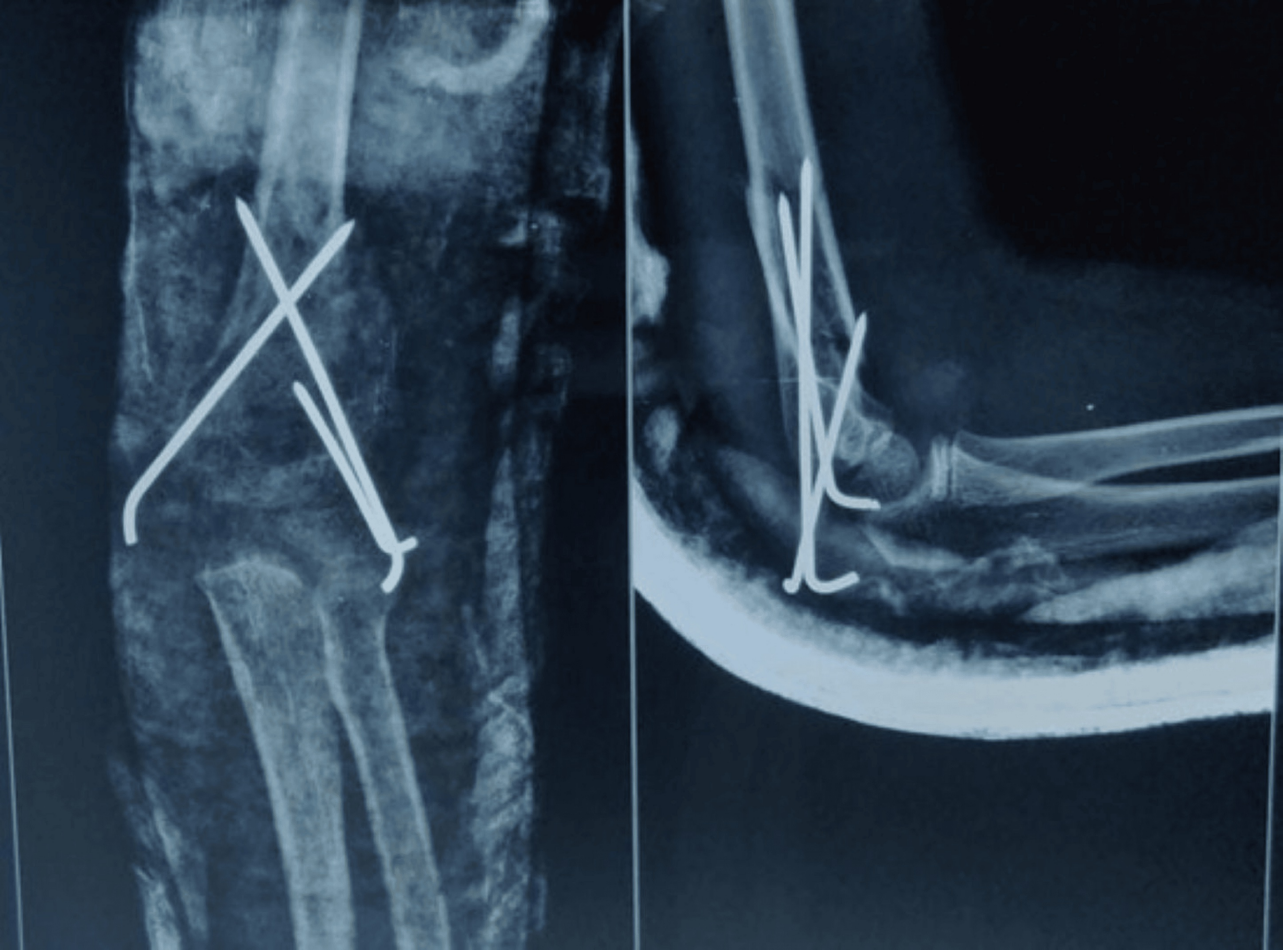
Postoperative radiograph of anteroposterior and lateral views, showing anatomical reduction of the fracture and proper placement of the pins.

An above-elbow slab was applied in a 45-degree extension, and was admitted to the hospital for surgical intervention. After necessary haematological investigations, the patient was operated on with closed reduction and crossed pinning with K-wires within 12 hours of admission. The procedure was done under general anaesthesia and all aseptic precautions. Multiple attempts for reduction and pinning were done to achieve anatomical reduction. The fracture was fixed with three (2.0 mm) K-wires, two medial and one lateral, and reduction, implant placement, and stability were confirmed under image intensification. The above-elbow slab was reapplied. Postoperative radiographs were taken to confirm the accurate pin placement and anatomical reduction of the fracture (Figure 2).

**Figure 3:**
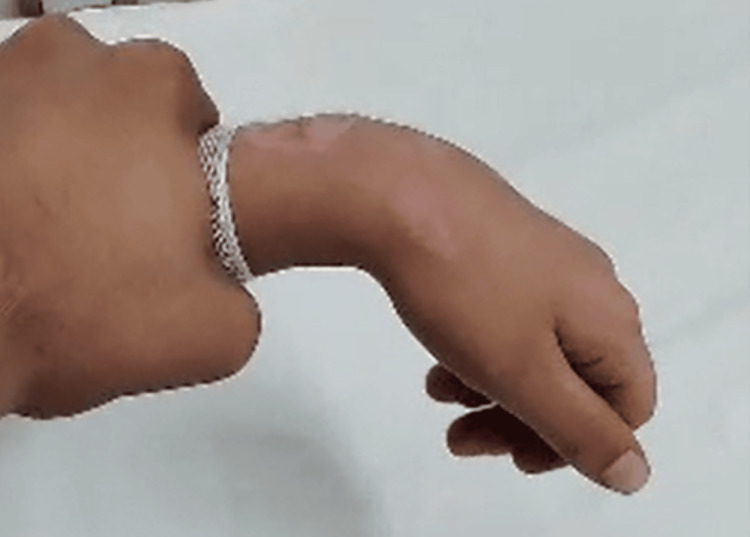
Immediate postoperative clinical image showing wrist drop.

Postoperatively, the patient had normal radial pulsation and distal vascularity, but the patient was unable to extend the wrist and fingers and was unable to abduct the thumb, with hypoesthesia over the dorsal surface of the hand and forearm (Figure 3).

**Figure 4:**
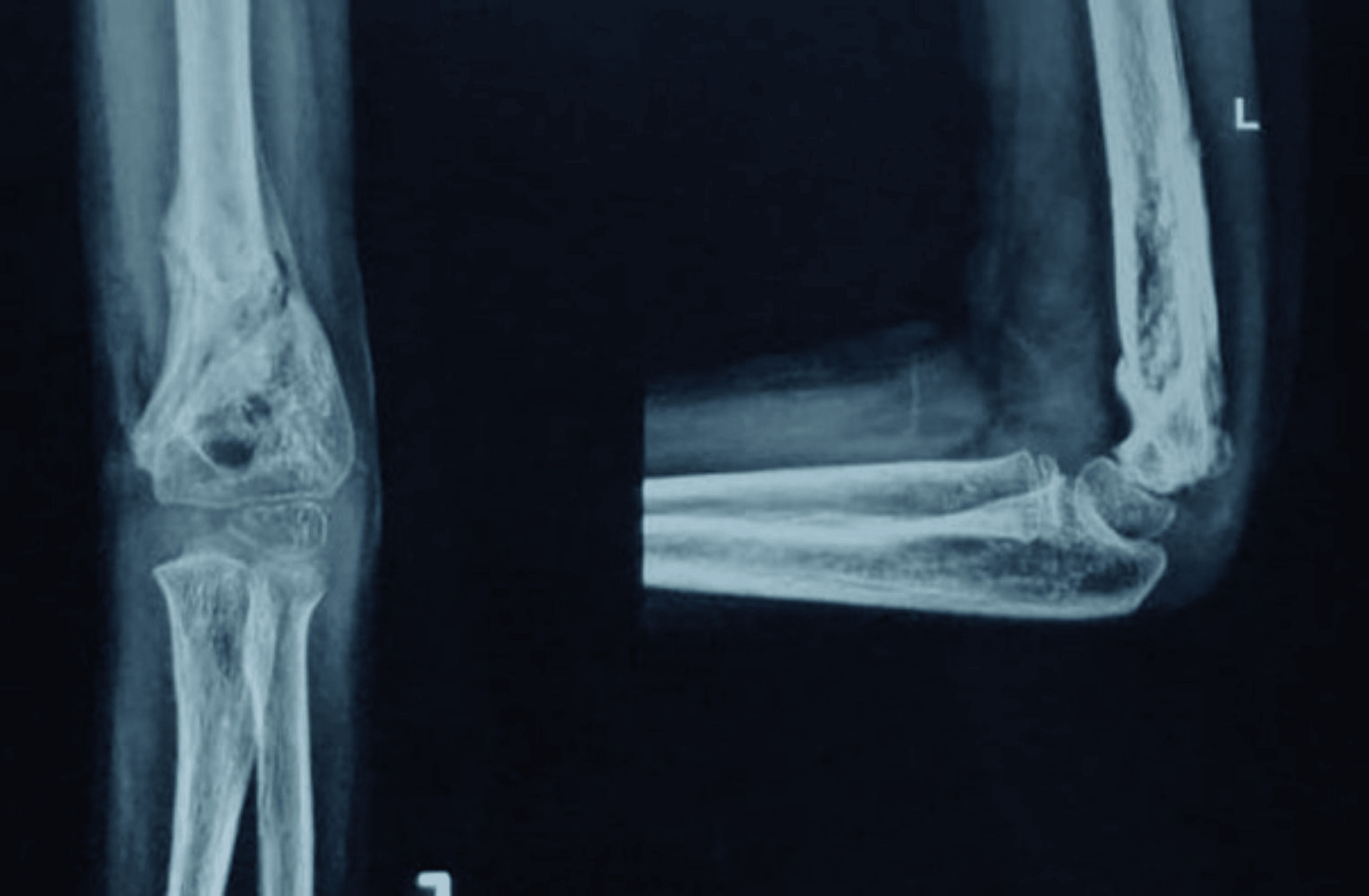
Radiograph of left elbow. Anteroposterior and lateral views after six weeks - implant removal, showing union of fracture.

A follow-up neurological examination in 24 hours found radial nerve weakness but with normal distal vascularity. The neurological deficit was managed conservatively with observation for four to six weeks. The patient was kept admitted for six days for observation for compartment syndrome and any signs of neurological improvement, but no neurological improvement was observed during the course of the hospital stay. The patient was discharged with an above-elbow slab and was advised to do regular follow-up. The patient came for regular visits every two weeks for six weeks, and a gradual improvement in wrist extension was seen. Pins were removed six weeks after the surgery, and radiographs were taken, which showed a well-united fracture (Figure 4).

**Figure 5:**
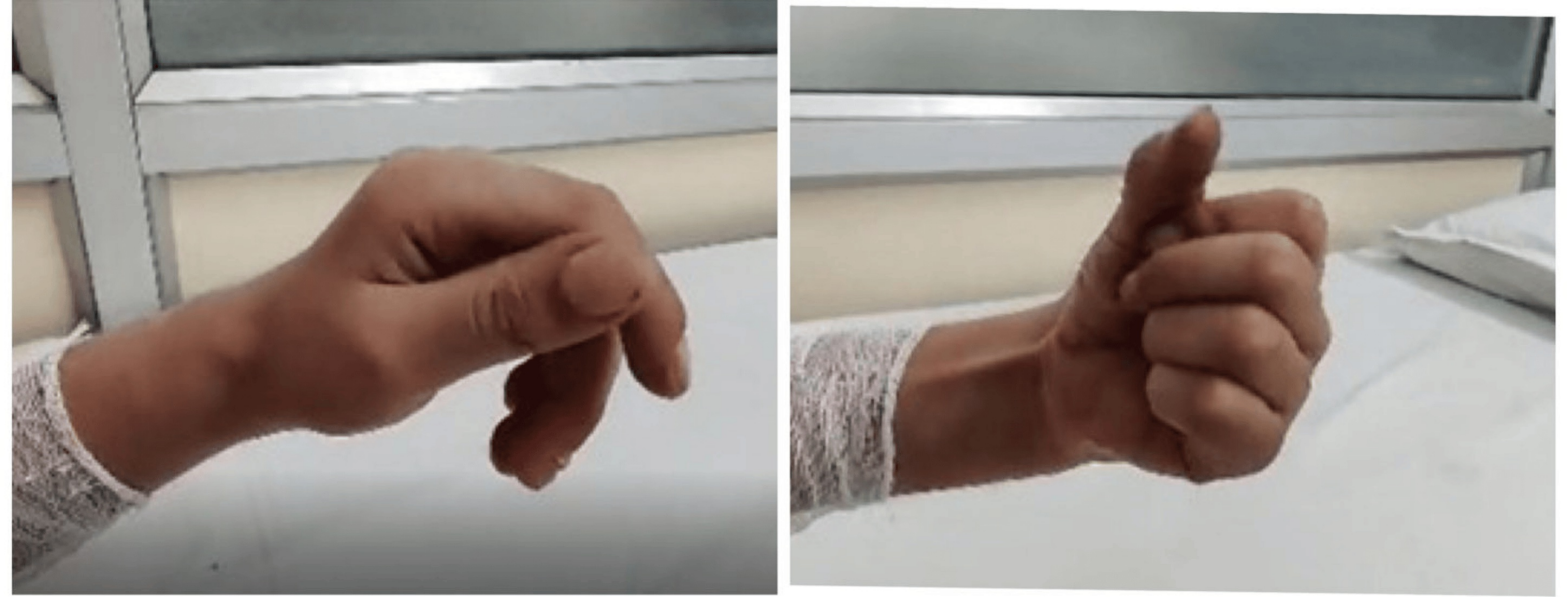
Improvement of thumb abduction and extension, and wrist extension at the end of six weeks.

Gentle elbow range of movements, including flexion, extension, supination, and pronation, was started after the K-wire removal and slab removal. Extension of the wrist joint was completely recovered, but fingers and the thumb showed partial improvement. Sensory function showed partial recovery, and there was improvement in thumb extension and abduction at six weeks (Figure 5).

**Figure 6:**
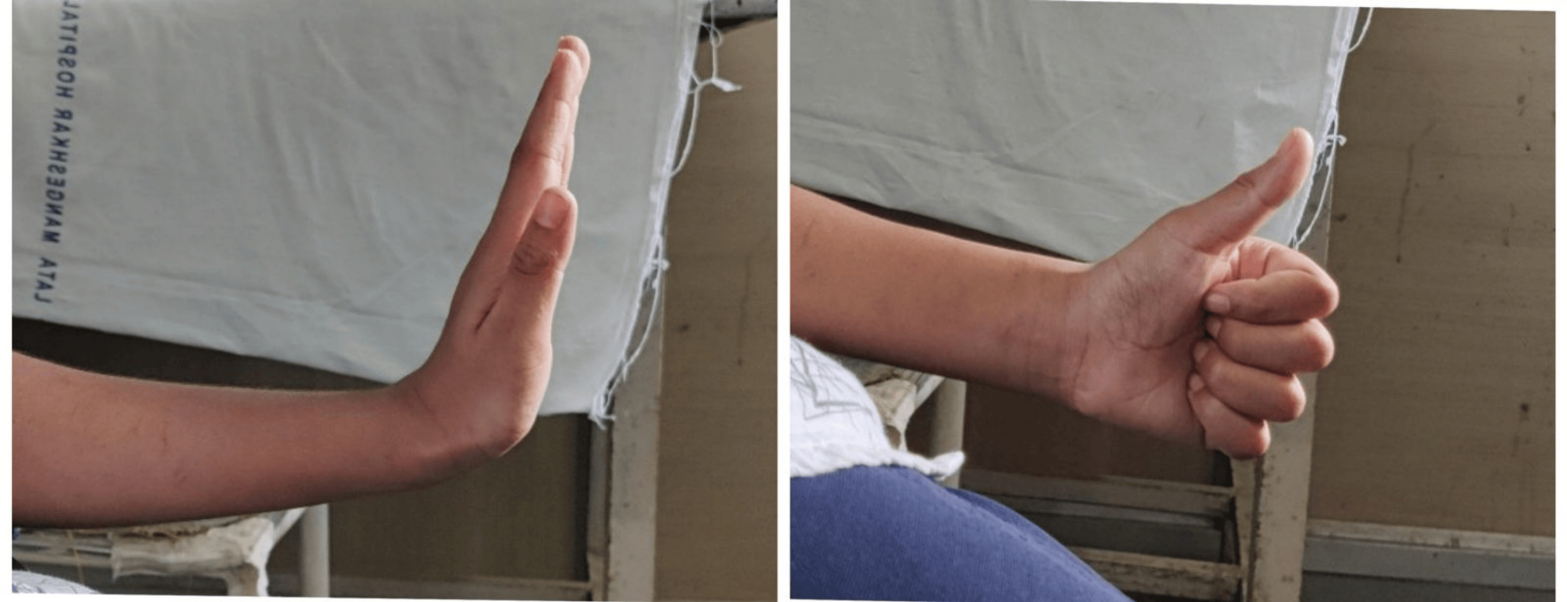
Complete wrist extension and thumb abduction at the end of eight weeks.

Further improvement in the form of complete wrist extension and thumb abduction was seen two weeks later at the end of eight weeks (Figure 6).

**Figure 7:**
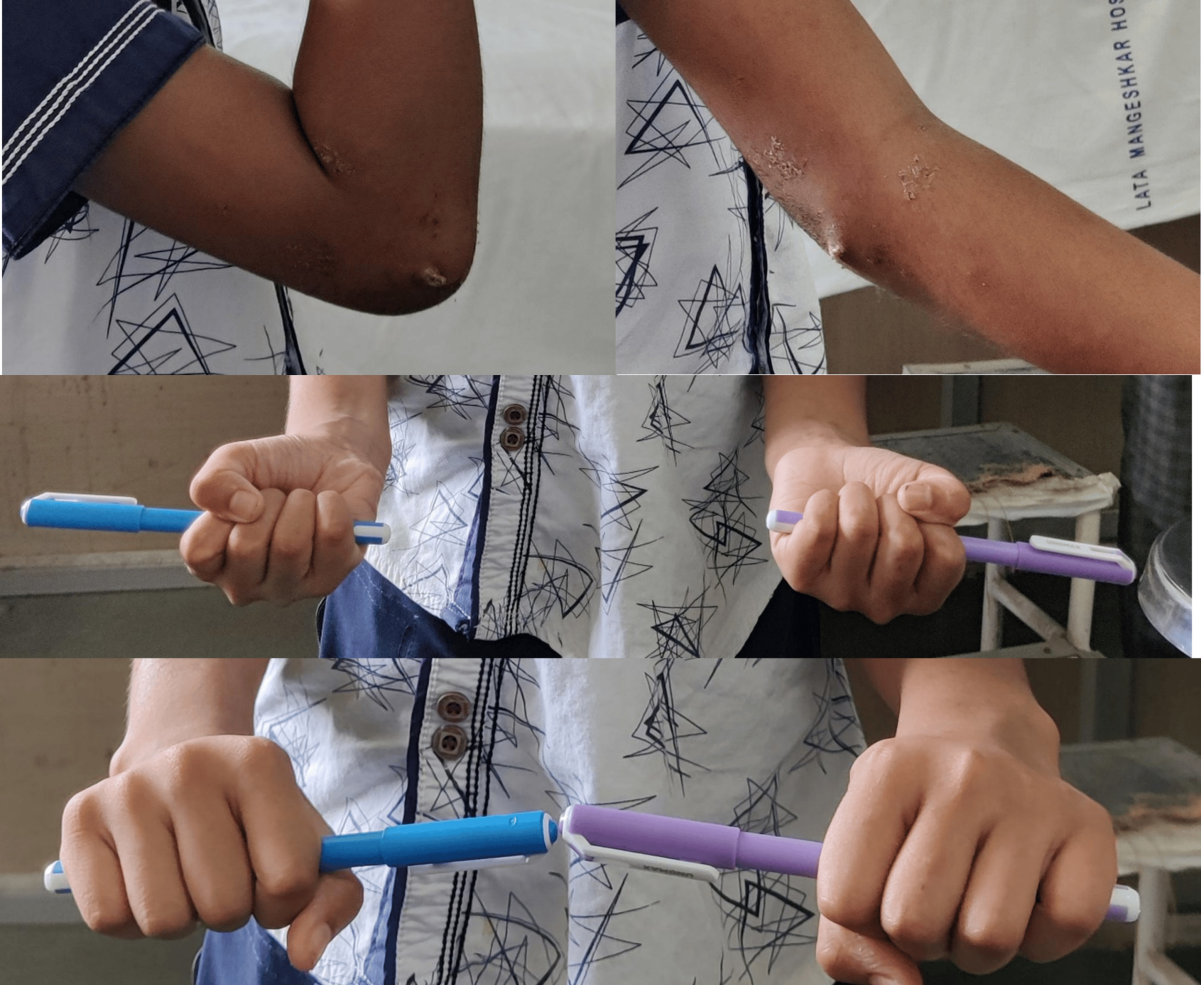
Good range of movements at the elbow at eight weeks.

Good elbow range of movements, including flexion, extension, supination, and pronation, was achieved after eight weeks (Figure 7).

Timeline

At the time of presentation, the patient had a fall on an outstretched hand while playing and complained of pain and deformity of the left elbow. On examination, there was swelling and restricted movement of the elbow; the neurovascular examination was normal. Radiographs confirmed a comminuted supracondylar humerus fracture. Closed reduction and crossed K-wire fixation were performed under general anaesthesia. Reduction and implant placement confirmed under image intensification.

In the immediate postoperative period, the radial pulse and distal vascularity were assessed and found to be normal. A neurological deficit was observed, including wrist drop, inability to extend the fingers, inability to abduct the thumb, and hypoesthesia over the dorsal surface of the hand and forearm.

On postoperative day 1, neurological examination remained unchanged, and compartment syndrome was excluded (intact radial pulse, no stretch pain), and we decided to manage conservatively with close observation.

On days 1-6, the patient was monitored for compartment syndrome and neurological recovery; however, no neurological improvement was observed during admission. The patient was discharged with an above-elbow slab.

In the second week, during the first follow-up, a gradual improvement in wrist extension was noted.

In the fourth week, during the second follow-up, the patient showed continued improvement in sensory function and further improvement in wrist extension.

In the sixth week, during the third follow-up, K-wire removal was performed, and radiographs confirmed fracture union, with complete recovery of wrist extension. Partial improvement in finger and thumb extension was also noted.

In the eighth week, during the fourth follow-up, we observed complete recovery of wrist extension and thumb abduction, along with full restoration of radial nerve function. A good elbow range of motion was achieved across all planes. Hence, no surgical exploration was required.

## Discussion

Injury to nerves is very commonly encountered following supracondylar humerus fractures, with the most common being the radial nerve. The extension type of supracondylar fractures is usually associated with posterior interosseous nerve (34%) injury, whereas the flexion type involves the ulnar nerve (91%). Iatrogenic neurological injury in closed or open reduction and K-wire fixation accounts for around 3-6% in supracondylar humerus fracture fixation [4].

The origin of the radial nerve is from the posterior cord of the brachial plexus, C5-T1 nerve roots, and exits the axilla to the posterior compartment of the arm. It then divides into a sensory branch and a branch supplying the long and medial head of triceps. Into the spiral groove of the humerus, it turns from medial to lateral, which makes the nerve very prone to humerus shaft fracture injuries. Then it divides into the lateral cutaneous nerve of the arm and the posterior cutaneous nerve of the forearm, and a branch to the anconeus muscle. The radial nerve then supplies the brachioradialis and extensor carpi radialis longus while piercing the lateral intermuscular septum, 7 to 10 cm above the lateral epicondyle [5]. It is important to know the course of the radial nerve to ascertain the level of trauma to the nerve. Most neurological injuries are neuropraxic and resolve spontaneously.

The sharp bone fragments in cases of supracondylar fractures usually cause the nerves to get entrapped and cause neurological deficits. Intraoperative injuries are usually caused by aggressive and multiple attempts to anatomically reduce the fracture during pin insertion. The ulnar nerve is most commonly affected in such cases, most commonly with crossed K-wire fixation. Complete recovery of nerve function is usually observed after K-wire removal and conservative management [6].

Our case had no preoperative neurological deficit. However, postoperatively, the patient had the absence of dorsiflexion of the wrist and fingers (wrist drop) and sensory deficit along the distribution of the radial nerve, which indicates high radial nerve injury. Postoperative radiographs were done, which showed the accurate position of wires and anatomical reduction of the fracture. On further examination, compartment syndrome was excluded as the patient had a good radial pulse and no positive stretch pain. After thorough clinical and radiological examination, we planned to avoid immediate surgical intervention and advised conservative management in the form of regular follow-ups and a thorough examination at each follow-up. The patient was called for regular follow-up every two weeks after discharge from the hospital. If improvement was not found to be as expected, we had decided to plan for surgical exploration of the nerve. Some studies showed a good result with a conservative approach [7]. Further investigations include CT scans and MRI scans, along with electrodiagnostic studies, which give an all-around approach for the management of these cases. Electrodiagnostic studies help to differentiate between high-grade and low-grade nerve injury, but other factors also play a role in nerve conduction studies and electromyography; hence, the exact location of the nerve injury determined by the above studies is questionable [8]. In most cases, conservative treatment for three to four months is given before taking the decision of surgical exploration.

Our patient was on regular follow-up every two weeks, and examination showed gradual improvement in sensory function with improvement in wrist extension at four weeks. Complete recovery of nerve function was observed at eight weeks post-surgery.

In our case, we found that multiple reduction attempts intraoperatively and multiple reduction manoeuvres and pinning may have caused neurological trauma to the radial nerve and being a neuropraxic injury, the recovery is complete after conservative management.

The electrophysiological study after eight weeks of the right upper limb is within normal limits. Nerve conduction velocities, distal latencies, and amplitudes for the radial nerve are appropriate for the patient’s age. Electromyography demonstrates no evidence of active denervation or chronic reinnervation changes in any radial nerve-innervated muscle, including those supplied proximal to the elbow.

## Conclusions

Iatrogenic radial nerve injury following surgical management of supracondylar humerus fractures is a well-recognised but uncommon complication, and postoperative high radial nerve palsy specifically represents an exceptionally rare occurrence in the literature. Based on existing evidence, iatrogenic neurological injury in this setting is most commonly partial in nature, typically attributable to neurapraxia resulting from stretching of the nerve during forceful or repeated reduction manoeuvres, or from mechanical irritation during multiple pinning attempts - rather than from direct transection.

The clinical course observed in this patient, namely, spontaneous and complete recovery of radial nerve function without surgical intervention, is consistent with the natural history of neurapraxic injuries as described in the broader literature. While conclusions cannot be drawn from a single case, this outcome aligns with the established consensus that the majority of postoperative neurological deficits in this context resolve conservatively. Surgical exploration is generally reserved for cases in which no clinical or electrophysiological improvement is demonstrated over a period of three to four months, a threshold this patient did not reach.
